# Nonlinear optical response of strain-mediated gallium arsenide microwire in the near-infrared region

**DOI:** 10.1515/nanoph-2023-0948

**Published:** 2024-03-13

**Authors:** Xiangpeng Cui, Wenjun Huo, Linlu Qiu, Likang Zhao, Junjie Wang, Fei Lou, Shuaiyi Zhang, Vladislav Khayrudinov, Wing Yim Tam, Harri Lipsanen, He Yang, Xia Wang

**Affiliations:** Shandong Engineering Research Center of New Optoelectronic Information Technology and Devices, School of Mathematics and Physics, Qingdao University of Science & Technology, Qingdao 266061, China; Department of Electronics and Nanoengineering, Aalto University, Espoo FI-00076, Finland; Department of Physics and William Mong Institute of Nano Science and Technology, Hong Kong University of Science and Technology, Clear Water Bay, Kowloon, Hong Kong, China; School of Instrumentation and Optoelectronic Engineering, Beihang University, Beijing 100191, China; School of Physics and Technology, University of Jinan, Jinan 250022, China

**Keywords:** nonlinear optical absorption, optical switch, GaAs microwire, strain-mediated

## Abstract

Gallium arsenide (GaAs) semiconductor wires have emerged as potent candidates for nonlinear optical devices, necessitating bandgap engineering for an expanded operational wavelength range. We report the successful growth of strain-mediated GaAs microwires (MWs) with an average diameter of 1.1 μm. The axial tensile strain in these wires, as measured by X-ray diffraction and Raman scattering, ranges from 1.61 % to 1.95 % and from 1.44 % to 2.03 %, respectively. This strain condition significantly reduces the bandgap of GaAs MWs compared to bulk GaAs, enabling a response wavelength extension up to 1.1 μm. Open aperture Z-scan measurements reveal a nonlinear absorption coefficient of −15.9 cm/MW and a third-order magnetic susceptibility of −2.8 × 10^−8^ esu at 800 nm for these MWs. I-scan measurements further show that the GaAs saturable absorber has a modulation depth of 7.9 % and a nonsaturation loss of 3.3 % at 1050 nm. In laser applications, GaAs MWs have been effectively used as saturable absorbers for achieving Q-switched and dual-wavelength synchronous mode-locking operations in Yb-bulk lasers. These results not only offer new insights into the use of large diameter semiconductor wires but also expand the potential for applications requiring bandgap tuning.

## 1 Introduction

Semiconductor wires have served as versatile building blocks for various optoelectronic devices. Thus far, semiconductor wires have found application in solar cells [1], [2], [3], [4], optical sensors [5], [6], and optical switches [7], among others. Due to their appealing characteristics, such as a direct bandgap, high electron mobility, and broad tunable bandgap, III–V semiconductor wires have attracted significant research attention. A notable example within this category is gallium arsenide (GaAs) wire, which constitutes a binary compound semiconductor composed of gallium from the III group and arsenic from the V group. GaAs, in its bulk form, possesses a bandgap of 1.424 eV at room temperature [8], [9], a density of 5.3176 g/cm^3^, and a maximum electron mobility [10] of 8500 cm^2^ V^−1^. Achieving defect-free growth of zinc blende bulk GaAs remains a challenge, particularly when not grown on GaAs substrates due to GaAs’s relatively large lattice constant of 5.65 Å [11].

One-dimensional semiconductor wires offer flexibility in substrate selection. Compared to their bulk counterparts, they can effectively alleviate strain energy at heterostructure interfaces, reduce defects, and facilitate defect-free growth of highly mismatched materials [12], [13]. However, it is essential to consider the decrease in thermal conductivity as the wire size decreases. As the size approaches or falls within the range of the phonon mean free path, the thermal conductivity significantly diminishes due to heightened scattering at boundaries [14], [15], [16]. This increased scattering primarily arises from the wire’s relatively small size, which approximates or falls below the phonon mean free path, leading to substantial phonon interference during propagation. Consequently, the thermal conductivity of nanowires generally registers lower values compared to bulk materials. Nevertheless, for large-diameter semiconductor wires (e.g., >200 nm), this phenomenon may be less pronounced (bulk mean free path: 117 nm [17]), and the effect of boundary scattering is expected to be minor. It is reasonable to propose that large-diameter GaAs wires exhibit a high thermal damage threshold.

Furthermore, with an increase in diameter, the aspect ratio of semiconductor wires notably decreases. This reduction significantly mitigates agglomeration during the liquid phase transfer process in device preparation, such as for saturable absorber (SA) mirrors, enhancing the reproducibility and performance of device fabrication. Simultaneously, the introduction of strain can effectively modulate the properties of semiconductor wires, including the bandgap, carrier mobility, and thermal conductivity, thus expanding the operational wavelength range and potential applications of these devices. Increasing the diameter of semiconductor wires positively influences their mechanical strength, enabling them to withstand greater stresses and strains. Previous reports have indicated that semiconductor wires can adjust their bandgap through strain, achieving reductions of up to 40 % [18]. GaAs, in particular, demonstrates superior mechanical elasticity, facilitating the tuning of bandgaps through tensile or compressive strain [19]. As previously mentioned, large-diameter III–V semiconductor wires may offer advantages in specific optoelectronic device applications.

To date, the predominant focus of research has centered on the utilization of small-scale wires (diameter < 100 nm) in electronic devices, including integrated circuits [20], [21], [22], [23] and photodetectors [24], [25], [26]. There has been a paucity of investigations into “one-dimensional” structures with diameters exceeding 200 nm, particularly in the context of third-order nonlinear optical responses and ultrafast optical modulation. Consequently, the exploration of the nonlinear optical properties of GaAs microwires (MWs) and their potential applications, such as in laser devices, assumes profound significance.

In this study, strain-mediated zinc-blende structure GaAs MWs were successfully synthesized via a self-catalyzed approach employing metalorganic vapor phase epitaxy, yielding an average diameter of 1.1 µm. The nonlinear optical absorption characteristics and third-order magnetic susceptibility of GaAs MWs were investigated utilizing Z-scan technology. Furthermore, GaAs MWs were employed as an efficient optical switch in a Yb-bulk laser, enabling the generation of Q-switched and ultrafast pulses. These findings underscore the promising prospects for large-diameter GaAs MWs in the domains of optoelectronic switches and other optical nonlinear responses.

## 2 GaAs MWs preparation and characterization

**Figure 1:**
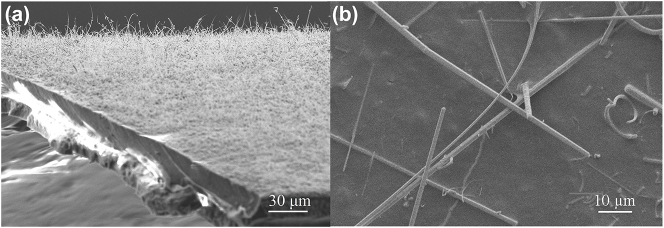
SEM images of GaAs MWs. (a) SEM image of GaAs MWs with a 30 µm scale. (b) SEM image of GaAs MWs with a 10 µm scale.

High-density GaAs MWs were synthesized on a flexible plastic substrate utilizing a horizontal atmospheric metalorganic vapor phase epitaxy system [27], [28]. Figure 1(a) illustrates the GaAs MWs grown on the flexible plastic substrate. Surface morphology analysis was performed using a scanning electron microscope (SEM, Zeiss Supra 40). The GaAs MWs exhibited a uniform and closely packed growth pattern with slight bending, indicative of high-quality semiconductor wires. These grown GaAs MWs could be readily detached from the plastic substrate by gently tapping and then transferred onto a quartz substrate using the vacuum filtration method. Figure 1(b) depicts typical GaAs MWs with an average diameter of approximately 1.1 μm. In this work, the SA was fabricated with a GaAs MW forest, which is not a true film. Consequently, the thickness of the GaAs SA is difficult to define. Based on the SEM image of GaAs samples, GaAs MWs (1–3 wires) are vertically stacked with a height in the range of 1–4 μm.

**Figure 2:**
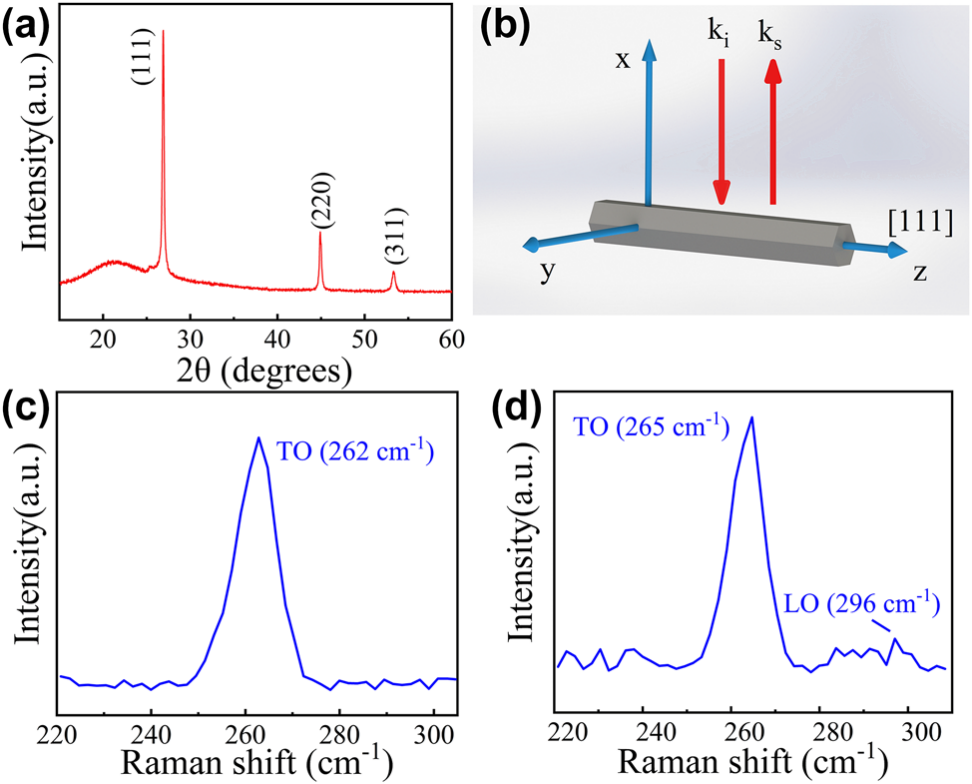
Characterization of GaAs MWs. (a) XRD patterns of GaAs MWs; (b) Schematic drawing of the scattering geometry of the Raman measurement. (c) and (d) Raman spectrum of GaAs MWs.

Characterization and analysis of the GaAs MW samples were conducted through X-ray diffraction [29] and Raman spectroscopy. GaAs MW products underwent comprehensive analysis using an X-ray diffractometer (XRD, IXRF-Model 550i) equipped with CuKα radiation (*λ* = 1.54 Å). The scanning parameters were set with a scanning step of 0.02° and a scanning rate of 5°/min. Figure 2(a) displays the X-ray diffraction pattern, with Miller indices clearly outlined for each diffraction peak. The primary diffraction peaks in the spectrum were observed at 26.77°, 44.86°, and 53.23°, corresponding to the (111), (220), and (311) crystallographic planes, respectively, indicative of the characteristic GaAs zinc-blende structure (JCPDS No. 89-2770) [30]. The prominent presence of the (111) peak signifies a pronounced texture in this crystallographic direction. The concentration ratio of unit cells aligned along the growth direction was discerned through the relative intensity of XRD peaks. GaAs MWs displayed relative peak intensities distinct from those found in standard reference data. Specifically, the relative intensity ratio for the (111), (220), and (311) unit cells in the reference data is 1:0.59:0.32, whereas the GaAs MWs exhibited a ratio of 1:0.26:0.11. This suggests a prevalence of (111) crystallographic units, indicating a preference for GaAs MWs to grow with the [111] orientation along the growth direction, while the side surfaces belong to the {110} crystal plane family [31], [32], [33].

Interestingly, a shift in the XRD peak toward smaller diffraction angles was observed, suggesting that the distance between adjacent lattice planes along the growth direction exceeded that of strain-free GaAs. This observation implies that the semiconductor wires experienced tensile strain, leading to alterations in lattice parameters. The correlation between strain and lattice modifications can be summarized as follows: tensile strain results in an increase in the interplanar distance of lattice planes. This relationship can be expressed as follows [18], [31]:

(1) $$\varepsilon_{z}=\left(\alpha_{z}-\alpha_{0}\right)/\alpha_{0}$$

where *α*_0_ = 5.6535 Å is the lattice parameter of strain-free GaAs. By comparing the (111) diffraction peak of GaAs MWs in XRD with that of bulk GaAs, we can use Bragg’s law to calculate the lattice constant along the growth direction [34], [35], which is *α*_*z*_ = 5.7635 Å. The strain along this direction (*ε*_*zz*_) has been estimated at 1.95 % using formula (1). Furthermore, we analyzed multiple sets of XRD data and the calculated lattice constants along the [111] direction fluctuated between 5.7445 Å and 5.7635 Å. Consequently, the actual axial strain likely falls within the range of 1.61 %–1.95 %.

Raman spectra of GaAs MWs were acquired using a Raman spectrometer (Renishaw inVia). Measurements were conducted with a laser wavelength of 532 nm. Figure 2(b) illustrates the typical backscattering geometrical configurations employed for polarized Raman measurements. GaAs MWs with a zinc-blende structure grew along the [111] direction and exhibited a hexagonal cross-section with crystal facets. In this backscattering geometry, the incident laser wave vector *k*_*i*_ aligned parallel to the *x*-axis, while the scattered wave vector *k*_*s*_ was oriented opposite to *k*_*i*_, ensuring alignment of the long axis of the semiconductor wire with the *z*-axis of the reference system. The linear polarization direction of the laser coincided with the *z*-axis, utilizing an $x\left(zz\right)\bar{x}$ polarization configuration. Figure 2(c) and (d) presents two typical Raman spectra obtained in our experiments. Figure 2(c) reveals a peak at 261 cm^−1^, corresponding to the transverse optical (TO) phonon mode. In this instance, the TO peaks exhibited a relatively small full-width at half-maximum of approximately 10 cm^−1^. The absence of longitudinal optical (LO) phonon modes in this configuration can be attributed to Raman selection rules [36], [37]. In backscattering geometry from the {110} crystal planes, the scattering of LO phonons is forbidden, allowing only TO phonon scattering. In Figure 2(d), both the TO phonon mode and a weak peak at 291 cm^−1^ are observed. The appearance of this additional peak can be attributed to two potential factors: 1) The presence of small (111) facets at the end of the GaAs MWs can result in the emergence of the LO phonon mode [38]. 2) Surface roughness may relax the selection rules, allowing for the observation of scattering by LO phonons. Previous studies have also established a correlation between the strength of the forbidden LO mode and the density of stacking faults. The absence or weakness of the LO mode suggests that the GaAs MWs used are of high quality [39].

Additionally, the observed peak exhibited a certain leftward shift, attributed to the tensile strain present in the GaAs nanowires. To estimate the axial strain in GaAs nanowires, a linear relationship between the relative energy leftward shift of TO phonons and the axial strain *ε*_*zz*_ within the semiconductor nanowire can be applied. This relationship can be expressed mathematically using a set of equations [40], [41]:

(2) $$H=\frac{1-2\nu }{3}$$

(3) $$\varepsilon_{zz}=\frac{\Delta \omega_{TO}}{\omega_{TO}}\frac{1}{\left[-3\gamma_{T}H+\gamma_{T}^{\prime }\left(1-H\right)\right]}$$

where *ω*_*TO*_ is the relaxed phonon frequency (in cm^−1^) of the optical (TO) mode phonons in GaAs, while *ν* = 0.16 is the Poisson’s ratio for GaAs oriented along the (111) crystallographic direction. *γ*_*T*_ = 1.35 is the hydrostatic phonon deformation potential parameter, and *γ′*_*T*_ = −0.88 is the shear deformation potential for GaAs TO phonons. It is important to highlight that current research findings indicate only minor deviations between bulk materials and semiconductor wires [40], [41]. Consequently, we employed the same Grüneisen parameter and Poisson’s ratio as those applicable to bulk GaAs. Our calculations, based on multiple sets of measured Raman data, reveal that the strain within GaAs falls within the range of 1.44 %–2.03 %. The leftward shift of Raman peaks and the increase in lattice constants signify the presence of tensile strain. Consequently, the presence of tensile strain in GaAs MWs has been independently confirmed through two experimental methods.

**Figure 3:**
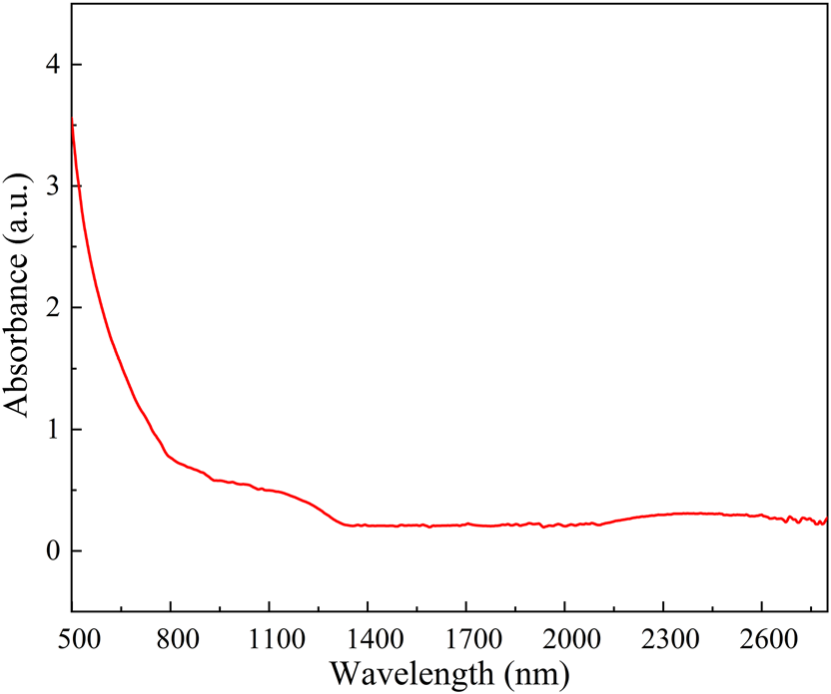
Absorption spectrum of GaAs MWs in the wavelength region of 500–2800 nm.

Literature research suggests that tensile strains ranging from 1.44 % to 2.03 % correspond to a bandgap change of approximately 0.12 eV–0.17 eV for small-diameter GaAs nanowires (it should be noted that the literature primarily focuses on nanowires with small diameters, and this trend may not strictly align with our findings [18], [41]). Given that key mechanical properties such as Young’s modulus can be diameter-dependent in semiconductor wires [42], confirming the change in bandgap for the GaAs MWs in this study requires additional experimentation. When the semiconductor wire diameter exceeds 1000 nm, exchange correlation effects may be underestimated in first-principles calculations. This could lead to significant errors in bandgap calculations. Furthermore, first-principles calculations for semiconductor wires typically have limitations for diameters exceeding 100 nm [43], [44], [45]. As the diameter increases, so does the system’s dimensionality, which can result in increased computational complexity. Therefore, we chose to conduct laser experiments instead of relying solely on theoretical calculations to validate the observed reduction in bandgap characteristics. Figure 3 shows the absorption spectrum in the wavelength region of 500–2800 nm, which indicates that the absorption edge of GaAs MWs exceeds 1100 nm.

## 3 Nonlinear optical properties of GaAs MWs

**Figure 4:**
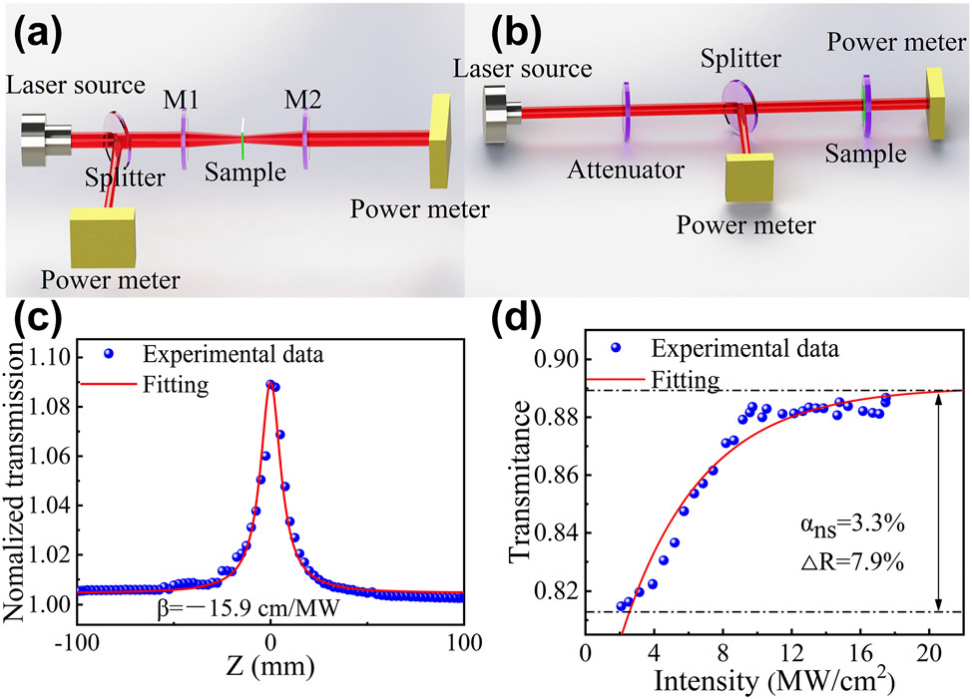
Nonlinear optical properties of GaAs MWs. (a) Schematic of the Z-scan experimental setup at 800 nm. (b) Schematic of the I-scan experimental setup at 1050 nm. (c) The open-aperture Z-scan data of GaAs MWs. (d) I-scan data of GaAs MWs.

To determine the nonlinear optical characteristics of GaAs MWs, we established experimental setups for both depth-dependent (Z-scan) and intensity-dependent (I-scan) measurements. These setups are illustrated in Figure 4(a) and (b), respectively.

The investigation of the nonlinear optical absorption properties of GaAs MWs was conducted through Z-scan experiments. GaAs MWs were dispersed in a cuvette containing acetone, with a concentration of approximately 0.8 mg/ml. To mitigate nonlinear effects stemming from the quartz cuvette and acetone, the GaAs MWs data were normalized, dividing the quartz cuvette with acetone as the solvent. In the open aperture Z-scan configuration, the sample was positioned on a motorized platform that oscillated at the focus of the focusing lens while maintaining constant incident laser power. This induced variations in the spot size incident on the sample, and the sample’s transmittance was recorded at different positions and under varying energy densities. The experimental measurements employed a Ti:sapphire laser (Tsunami, Spectra-Physics) with an 60 fs pulse width and a central wavelength of 800 nm. A peristaltic pump was used to flow the GaAs MWs continuously into the cuvette and avoid the possible cumulative thermal effect on the nonlinear response. The obtained data [Figure 4(c)] were fitted using the following formula [46]:

(4) $$T\left(Z\right)=1-\beta I_{0}L_{\mathrm{eff}}/\left[2^{\frac{3}{2}}\left(1+\frac{Z^{2}}{Z_{0}^{2}}\right)\right]$$

In the given equation, *β* represents the nonlinear saturation absorption coefficient of the sample and *I*_0_ represents the peak intensity at the lens focal point (*Z* = 0). *Z* is the relative position of the sample, and *L*_*eff*_ is the effective thickness. The peak at the center of the curve indicates the occurrence of saturation absorption of the GaAs MWs. In addition, Im*χ* ^(3)^ can be calculated using the following formula [47]:

(5) $$Im\chi^{\left(3\right)}=\frac{2\varepsilon_{0}c^{2}n_{0}^{2}}{3\omega }\beta$$

where *c* is the speed of light in vacuum, *n*_0_ is the linear refractive index, *ε*_0_ is the vacuum dielectric constant, and *ω* is the angular frequency. The nonlinear optical absorption coefficient *β*_*eff*_ and third-order magnetic susceptibility Im*χ* ^(3)^ of the GaAs MWs were determined to be −15.9 cm/MW and −2.8 × 10^−8^ esu, respectively.

In Figure 4(b), reflectance-based intensity scanning measurements were carried out using a self-constructed Yb:SYB continuous-wave laser, which was modulated into pulses using a chopper operating at a frequency of 1 kHz. A laser beam with a wavelength of 1050 nm was divided into two beams utilizing a beamsplitter, and two power meters were employed to record the power before and after traversing the GaAs MW at different incident power densities. The experimental data were subjected to fitting using the following equation [46], [48], [49], [50]:

(6) $$T\left(Z\right)=1-\frac{\Delta R}{1+\frac{I}{I_{s}}}-\alpha_{ns}$$

The formula employs Δ*R*, *I*, *I*_*s*_, and *α*_*ns*_ to symbolize the modulation depth, incident intensity, saturation intensity, and nonsaturable loss, respectively. Utilizing this formula with the measured data [Figure 4(d)], we derived the modulation depth, saturation intensity, and nonsaturable loss of GaAs at 1 µm, which amounted to 7.9 %, 4.69 MW/cm^2^, and 3.3 % (considering the Fresnel reflection loss of ∼8 % for both sides of the quartz substrate in this work), respectively. The modulation depth and saturation intensity of the GaAs-MW-SA were lower than those of other exemplary SAs reported, such as Bi_2_O_2_Te (8.2 %, 91.25 MW/cm^2^) [51], PbTe (10.4 %, 26 MW/cm^2^) [52], and hydrazone organics (39.04 %, 55.19 MW/cm^2^) [53]. The underlying principle can be elucidated by the Pauli exclusion principle. As the incident light intensity increases, electrons within the valence band progressively absorb energy and transition to excited states. This ultimately leads to saturation in light absorption as a majority of electrons become excited. However, due to the Pauli exclusion principle, which prohibits multiple electrons from occupying the same excited state, the material ceases to absorb the incident laser, resulting in a state of high transmittance [54].

As depicted in Table 1, large-diameter GaAs MWs exhibit noteworthy nonlinear absorption characteristics that are on par with those observed in previously wire structures. This position positions them as highly promising candidates for utilization as SAs in Q-switching and mode-locking solid-state lasers.

**Table 1:** The nonlinear optical parameters of wire-based saturable absorbers.

Wires	Average diameter (nm)	*λ* (nm)	*β*_*eff*_ (cm/MW)	Im*χ* ^(3)^ (esu)	*τ* (ps)	*I*_*s*_ (MW/cm^2^)	Δ*R* (%)	Ref.
InP	65	1064	−1.2 × 10^2^	−1.3 × 10^−7^		∼90	∼11	[55]
	65	800	–	–	8.1, 63.8	–	–	
InAs	100	1064	−1.0 × 10^5^	−2.2 × 10^−4^	2.07	0.13	16.7	[56]
	100	2000	−1.3 × 10^5^	−6.5 × 10^−4^	7.11	0.03	14	
	100	2800	−1.1 × 10^5^	−7.4 × 10^−4^	8.82	0.01	12.7	
InAsP	50	532	−2.1 × 10^2^	−2.6 × 10^−7^	–	∼800	∼33	[57]
	50	1064	−1.4 × 10^2^	−3.7 × 10^−7^	–	∼250	∼15	
	50	675	–	–	∼2, ∼15	–		
InSb	1200	800	−65.5 ± 0.33	(−1.96 ± 0.01) × 10^−7^	–	(7.77 ± 1.13) × 10^2^	6.77 ± 0.68	[58]
	1200	1050	−49.8 ± 0.14	(−1.95 ± 0.01) × 10^−7^	6.1, 53.2	(4.19 ± 0.41) × 10^2^	4.97 ± 0.19	
	1200	1900	(−8.5 ± 0.05) × 10^4^	(−6.02 ± 0.04) × 10^−4^	–	11.14 ± 0.44	4.47 ± 0.11	
	1200	2800	(−5.2 ± 0.02) × 10^4^	(−5.48 ± 0.03) × 10^−4^	–	7.08 ± 0.38	2.68 ± 0.01	
GaAs	1100	800	−15.9	−2.8 × 10^−8^	–	–	–	This work
	1100	1050	–	–	0.8, 43.7	4.69	7.9	

## 4 Laser application based on GaAs MWs

**Figure 5:**
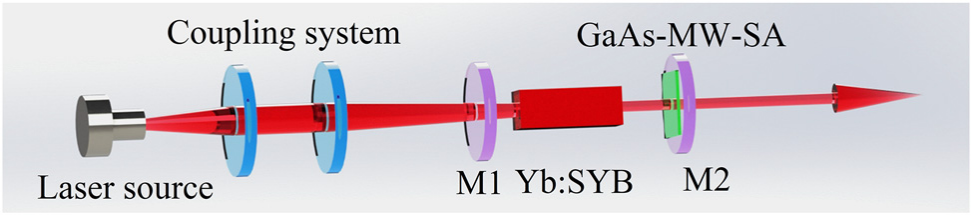
Configuration of the passively Q-switched laser based on GaAs-MW-SA.

To further investigate the absorption saturation property, passive Q-switching was achieved using GaAs-MW-SA within a Yb:SYB laser setup, as depicted in Figure 5. The optical components included an input mirror (M1) with high transmittance within the 900–990 nm range and high reflectivity within the 1010–1100 nm range and output mirrors (M2) with different transmittances (*T* = 1.5 and 4 %). A GaAs-MW-SA was integrated into the laser cavity. A fiber-coupled laser diode emitting at a central wavelength of 978 nm served as the pump source, with a fiber core diameter of 105 μm and a numerical aperture of 0.22. The pump beam was focused into the Yb:SYB crystal using a 1:1 focusing system. To safeguard the crystal, it was enveloped in indium foil and secured in a copper block maintained at a constant temperature of 13 °C during the experiment.

**Figure 6:**
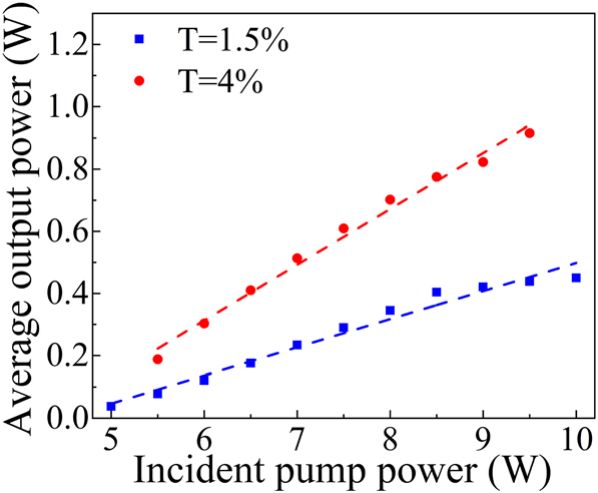
Average output power as a function of the incident pump power.

Figure 6 illustrates the relationship between the absorbed pump power and Q-switched average output power for different output coupling transmittances (*T* = 1.5 % and 4 %). At an output coupling transmittance of 1.5 %, the Q-switching laser threshold was determined to be 5 W, beyond which the average output power exhibited linear growth. At an incident pump power of 10 W, an average output power of 0.45 W was achieved, resulting in an optical-to-optical efficiency of 4.5 %. When the output coupling transmittance was set to 4 %, the Q-switching laser threshold remained at 5.5 W. Upon increasing the pump power to 9.5 W, the maximum average output power attained was 0.92 W, yielding an optical-to-optical efficiency of 9.6 %. Further increase in pump power led to instability in the Q-switching operation.

**Figure 7:**
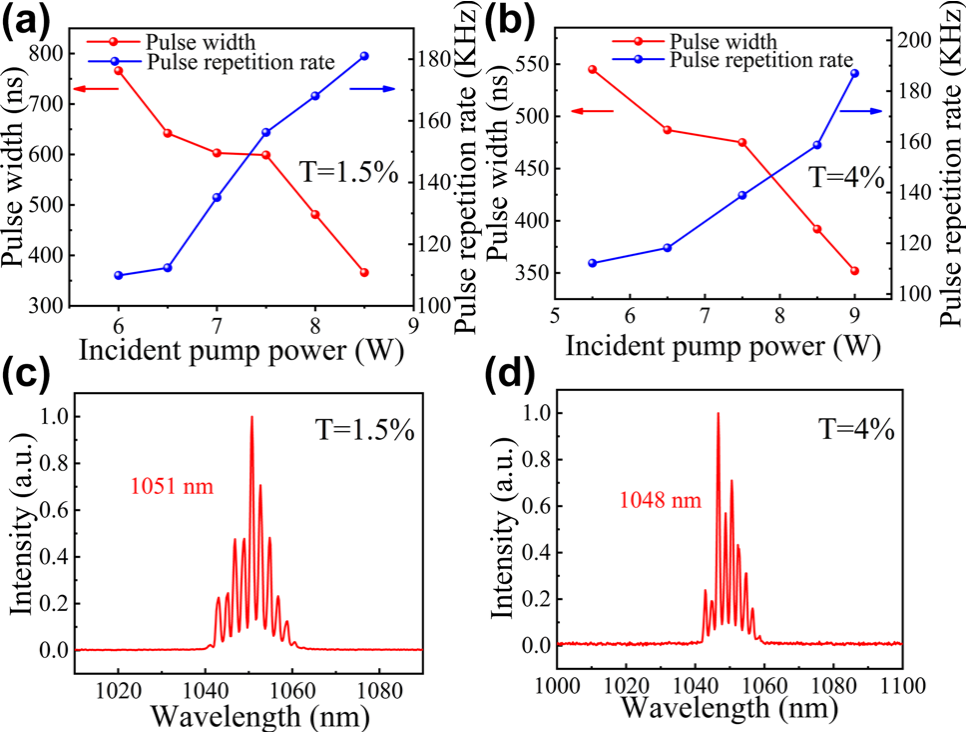
The Q-switched results of Yb-laser based on GaAs-MW-SA. (a) and (b) The variation of the pulse repetition rate and the pulse width as functions of the incident pump power for *T* = 1.5 % and 4 %, respectively. (c) and (d) Emission spectra of PQS lasers for *T* = 1.5 % and 4 %, respectively.

**Figure 8:**
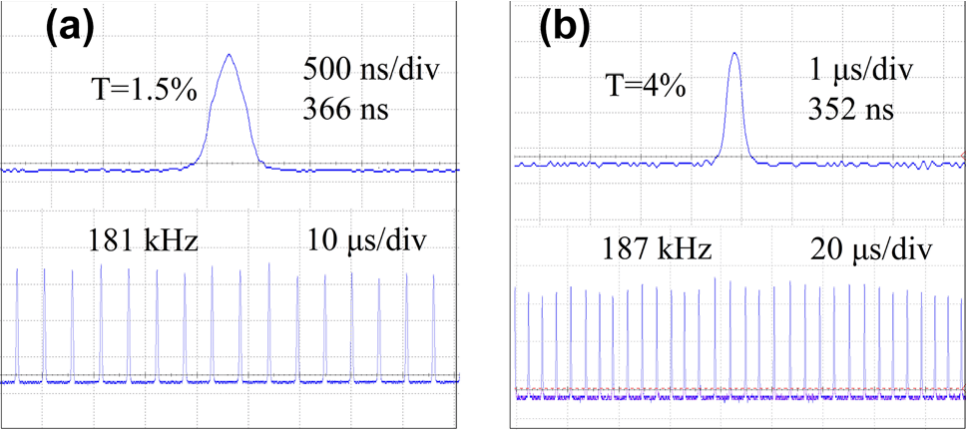
The recorded pulse profiles. (a) and (b) The shortest PQS pulse profiles and the corresponding pulse trains for *T* = 1.5 % and 4 %, respectively.

Figure 7(a) and (b) illustrates the pulse width and pulse repetition rate as functions of the incident pump power, recorded for the cases of *T* = 1.5 % and *T* = 4 % when the pulse profile was stable. Figure 7(c) and (d) depicts the corresponding laser emission spectra in each case. For *T* = 1.5 %, the pulse width decreased from 766 ns to 366 ns, and the pulse repetition frequency increased from 110 kHz to 181 kHz as the pump power ranged from 6 W to 8.5 W. For *T* = 4 %, the pump power ranged from 5.5 W to 9 W, resulting in a reduction in the pulse width from 545 ns to 352 ns and an increase in the repetition rate from 112 kHz to 187 kHz. Notably, the output coupling of *T* = 1.5 % exhibited a lower threshold than that of *T* = 4 %. For both *T* = 1.5 % and *T* = 4 %, the center wavelengths during stable Q-switched operation were 1051 nm and 1048 nm, respectively. This phenomenon can be attributed to the change in the peak wavelength of the Yb:SYB gain cross-section. As the transmittance of the output mirror increased, the proportion of upper-level population inversion also increased, resulting in a blueshift in the peak wavelength of the gain cross-section. Figure 8(a) and (b) depicts the pulse trains recorded when the shortest laser pulses were generated under each output coupling.

In the domain of nonlinear optoelectronic devices, a comprehensive understanding of the dynamic behavior of charge carriers within semiconductors is of primary importance. To investigate this behavior, we established a pump–probe setup employing a Ti:sapphire laser operating at a frequency rate of 80 MHz, with a pulse duration of 150 fs. The pump beam, centered at a wavelength of 800 nm, was utilized to excite the photocarriers. Varied time delays between the pump and probe pulses enabled the study of the dynamic behavior of charge carriers. Analysis of changes in the probe signal as a function of the pump–probe delay provides valuable insights into carrier dynamics, including their lifetimes. For the analysis of the experimental data, a double-exponential function was employed:

(7) $$\frac{\Delta T}{T_{0}}\left(t\right)=A_{1}e^{-t/\tau_{1}}+A_{2}e^{-t/\tau_{2}}$$

**Figure 9:**
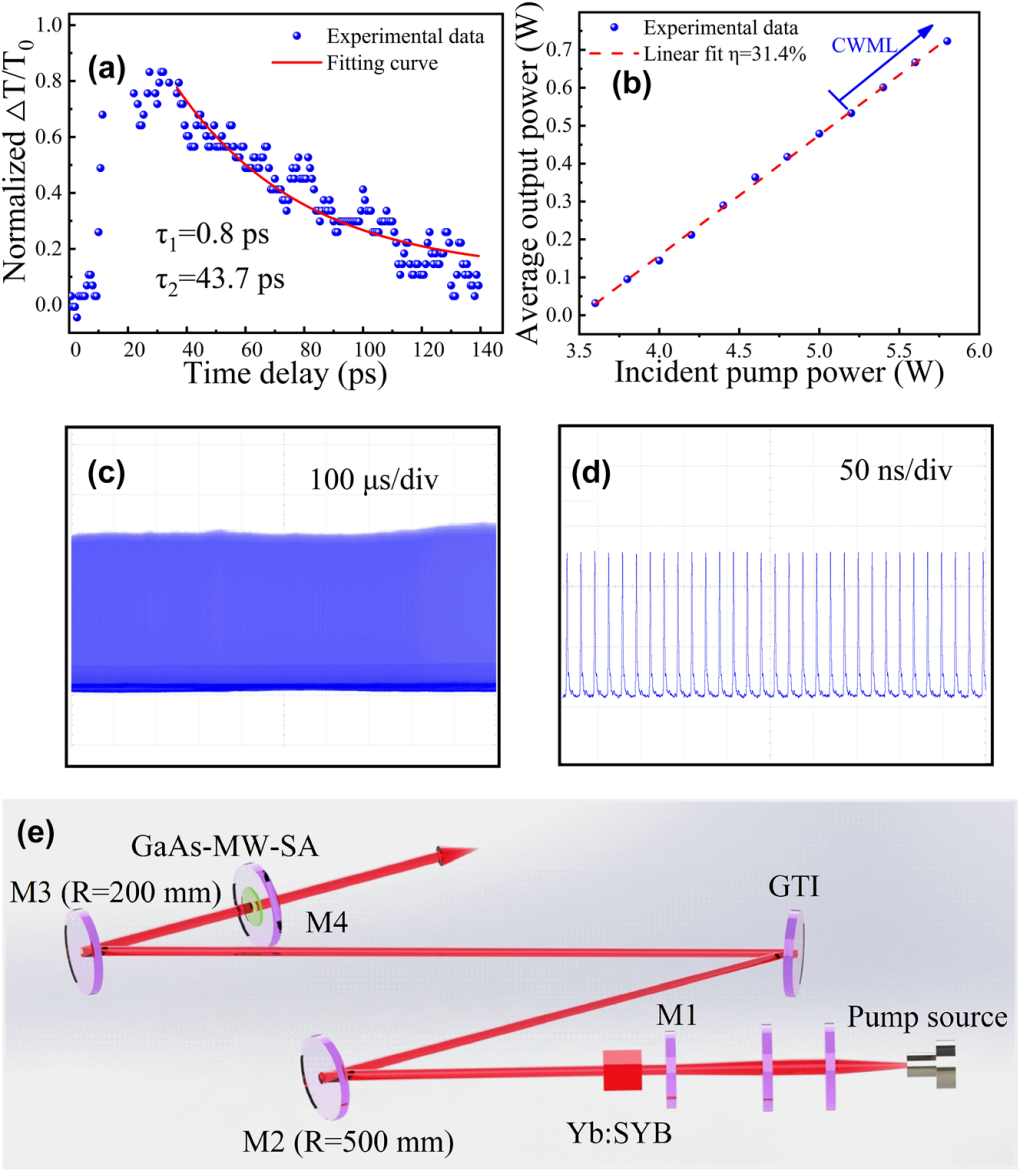
The pump-probe data and ultrafast laser applications of GaAs MWs. (a) Differential transmission of GaAs MWs with a 1050 nm probe laser. (b) Average output power as a function of the incident pump. (c) and (d) The mode-locked pulse train recorded with time scales of 100 μs/div and 50 ns/div, respectively. (e) Configuration of the mode-locked laser based on GaAs-MW-SA. M1, input mirror: flat mirror coated for HT at 900–990 nm and HR in 1010–1100 nm; M2 and M3, HR fold mirrors. GTI: HR mirror with dispersion of −250 (±50) fs^2^ per reflection; M4, output mirror with a transmittance of 1.5 % at 1020–1100 nm.

The relative change in probe beam transmission with and without the pump beam is denoted by Δ*T*/*T*_0_, where *T*_0_ represents the initial transmission. The typical relaxation times attributed to intraband carrier-phonon scattering and interband carrier recombination are represented by *τ*_1_ and *τ*_2_, respectively. The relative amplitudes of the dual temporal components are denoted by *A*_1_ and *A*_2_. *τ*_1_ and *τ*_2_ for GaAs-MW-SA were found to be 0.8 ps and 43.7 ps, respectively, as shown in Figure 9(a), and obtained through fitting experimental data. These results indicate the potential of GaAs MWs as an effective mode-locker.

The applications of ultrafast optics are expansive, covering various domains such as the production of Bessel-like terahertz waves via superluminal laser plasma filaments [59], the use of subpicosecond pulses for generating terahertz waves [60], the generation of high-order harmonics [61], investigations into optical Rogue waves [62], and the development of ultrafast laser systems [63]. The evaluation of an ultrafast laser based on GaAs-MW-SA took place within a folded cavity, as depicted in Figure 9(e). Through precise adjustments of the cavity mirrors, stable dual-wavelength synchronous continuous-wave mode-locking (CWML) operation was achieved at a pump power of 5.2–5.7 W, as illustrated in Figure 9(b).

In the CWML regime, Figure 9(c) and (d) displays a typical mode-locked pulse train with time scales of 100 µs and 50 ns, respectively. The laser consistently maintained a stable mode-locked state, which could endure for extended durations. The pulse repetition rate amounted to 62.5 MHz, corresponding to the 2.4 m W-shaped cavity. In our laser configuration, the length of the mode-locked cavity was approximately 120 times longer than that of the Q-switched laser cavity. Based on the passive mode-locking theory for solid-state lasers, the minimum average intracavity power of the current “Q-switched laser cavity” for achieving a CWML operation should be much higher than that of the mode-locked cavity [64]. Furthermore, multiple longitudinal modes can exist in a laser cavity, which are the stable oscillation modes of light waves along the axis of the resonator. As the cavity length decreases, the increased spacing between longitudinal modes necessitates a higher modulation frequency to lock these modes, imposing greater demands on the modulator’s performance. In this study, a CWML laser operation was only realized in the long folded cavity.

**Figure 10:**
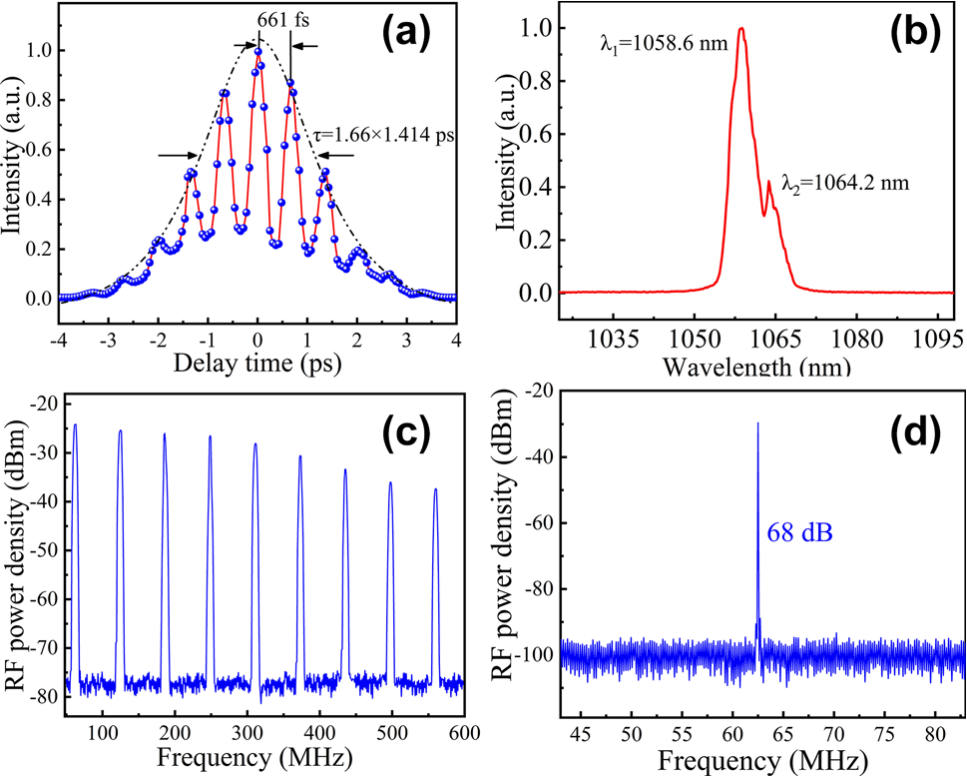
The mode-locked results of Yb-laser based on GaAs-MW-SA. (a) Autocorrelation trace of the mode-locked laser. (b) Laser spectrum of the mode-locked laser. (c) 600 MHz wide-span RF spectrum of the mode-locked laser. (d) RF spectrum at the fundamental beat note.

The autocorrelation trace, as measured using the APE PulseCheck SM 2000, is depicted in Figure 10(a), revealing a multipeak structure, indicative of dual-wavelength synchronous mode locking. Within the CWML regime, a stable intensity modulation pattern was discernible in the autocorrelation trace. The mode-locked pulses exhibited a pulse duration of 1.66 ps, assuming a Gaussian shape. The beat pulse exhibited a period of 661 fs, aligning with the dual-wavelength structure observed in the spectrum. As depicted in Figure 10(b), the dual wavelengths centered at 1058.6 nm and 1064.2 nm, with a frequency difference of 1.491 THz. Our laser operates at a wavelength of approximately 1060 nm, indicating that the strain induces a minimum reduction of 0.26 eV in the bandgap of GaAs MWs.

Figure 10(c) and (d) illustrates the RF spectrum, captured using the MXA Signal Analyzer N9020A, at various scales after the mode-locked laser had maintained stability for an hour. A high extinction ratio of 68 dB was detected at the fundamental beat note. Notably, in the previous study, the output from the Yb:SYB mode-locked laser consistently exhibited a single wavelength when employing SESAM as the mode-locking element [65]. However, in the current study, the mode-locking pulses manifest dual-wavelength synchronous mode locking. This observation suggests that GaAs MWs introduce an additional wavelength selection mechanism. In this work, the potential mechanism can be ascribed to level splitting arising from the presence of tensile strain in GaAs MWs, a consequence of the reduction in crystal symmetry [66]. When the incident laser incorporates waves of multiple frequencies, these waves may induce saturation effects on the absorber at distinct frequencies. This variance in the absorber’s response to light of varying frequencies can result in differing losses in the laser output at two or more frequencies, ultimately leading to the segregation of wavelengths and the emergence of dual-wavelength output. Synchronized multi-wavelength mode-locking has been observed in fiber lasers. Referring to related studies [67], [68], we can discern disparities in the synchronization of mode locking between solid-state lasers and fiber lasers. Solid-state lasers may capitalize on intrinsic material attributes, such as the energy level splitting observed in GaAs microwires, while fiber lasers rely on meticulous manipulation of dispersion within the cavity confines.

## 5 Conclusions

In summary, our characterization of GaAs MWs reveals the feasibility of applying strain to large-diameter semiconductors, rendering them exceptionally suitable for optoelectronic devices necessitating bandgap tuning. Raman and XRD measurements revealed tensile strain within the range of approximately 1.4 %–2 % in the GaAs MWs. This strain has led to a notable reduction in the bandgap, amounting to a substantial 18 % decrease. The nonlinear absorption properties of GaAs MWs were examined through Z-scan and I-scan measurements, revealing a nonlinear absorption coefficient of −15.9 cm/MW at 0.8 μm and a modulation depth of 7.9 % at 1.1 μm. In the context of laser applications, GaAs MWs have proven to be effective optical switches, facilitating the realization of Q-switched and dual-wavelength synchronous mode-locked bulk lasers operating within the near-infrared wavelength region. This study signifies a significant advancement in the utilization of large-diameter semiconductor wires for nonlinear optical devices, harnessing the influence of strain to modulate optical performance effectively.
